# A quantitative approach for trap analysis between Al_0.25_Ga_0.75_N and GaN in high electron mobility transistors

**DOI:** 10.1038/s41598-021-01768-4

**Published:** 2021-11-17

**Authors:** Walid Amir, Ju-Won Shin, Ki-Yong Shin, Jae-Moo Kim, Chu-Young Cho, Kyung-Ho Park, Takuya Hoshi, Takuya Tsutsumi, Hiroki Sugiyama, Hideaki Matsuzaki, Tae-Woo Kim

**Affiliations:** 1 Department of Electrical, Electronic and Computer Engineering, University of Ulsan, Ulsan, 44610 Korea; 2 Korea Advanced Nano Fab Center, Suwon, Gyeonggi-do 16229 Korea; 3 NTT Device Technology Laboratories, NTT Corporation, Atsugi, Kanagawa 243-0198 Japan

**Keywords:** Electrical and electronic engineering, Materials for devices, Nanoscale materials

## Abstract

The characteristics of traps between the Al_0.25_Ga_0.75_N barrier and the GaN channel layer in a high-electron-mobility-transistors (HEMTs) were investigated. The interface traps at the Al_0.25_Ga_0.75_N/GaN interface as well as the border traps were experimentally analyzed because the Al_0.25_Ga_0.75_N barrier layer functions as a dielectric owing to its high dielectric constant. The interface trap density *D*_*it*_ and border trap density *N*_*bt*_ were extracted from a long-channel field-effect transistor (FET), conventionally known as a FATFET structure, via frequency-dependent capacitance–voltage (C–V) and conductance–voltage (G–V) measurements. The minimum *D*_*it*_ value extracted by the conventional conductance method was 2.5 × 10^12^ cm^−2^·eV^−1^, which agreed well with the actual transistor subthreshold swing of around 142 mV·dec^−1^. The border trap density *N*_*bt*_ was also extracted from the frequency-dependent C–V characteristics using the distributed circuit model, and the extracted value was 1.5 × 10^19^ cm^−3^·eV^−1^. Low-frequency (1/*f*) noise measurement provided a clearer picture of the trapping–detrapping phenomena in the Al_0.25_Ga_0.75_N layer. The value of the border trap density extracted using the carrier-number-fluctuation (CNF) model was 1.3 × 10^19^ cm^−3^·eV^−1^, which is of a similar level to the extracted value from the distributed circuit model.

## Introduction

Recently, GaN-based high-electron-mobility-transistors (HEMTs) have gained considerable attention because of their outstanding material properties and device performance, including power and RF applications up to the sub-terahertz regime^1–3^. These advantageous properties and performance are attributable mainly to the high quality of the epitaxial layer comprising the Al_x_Ga_1−x_N barrier and the GaN channel layer, which causes the formation of two-dimensional electron gas (2DEG) on top of Si, Sapphire, and SiC substrates^4,5^. The quality of the Al_x_Ga_1−x_N/GaN interface is crucial to the improvement of the carrier transport in the channel during device operation^6,7^. In addition to the performance of AlGaN/GaN HEMTs, their reliability is an ongoing topic of research that requires examination of a variety of factors. Most of the reliability issues are related to the AlGaN layer, which is the surface layer in the gate-to-drain access region and contains deep-level traps^8^. These issues become more critical in deeply scaled transistors for high-frequency applications. Most previous research on the reliability of AlGaN/GaN HEMTs focused mainly on the surface traps in the access region and on their passivation using dielectric materials^9,10^. Plasma treatment was also utilized to decrease these deep-level traps in the AlGaN barrier layer^11^. Characterization of interface traps and deep-level border traps is important for improving the 2DEG carrier concentration and reducing interface roughness scattering to enhance mobility, with the eventual aim of improving device reliability and performance. Previous studies focused on the characterization of traps formed at the insulator/AlGaN interface in a metal–oxide–semiconductor field-effect transistor (MOSFET) structure via frequency-dependent capacitance–voltage (C–V) and conductance–voltage (G–V) measurements^9,10^. Some studies even discussed the interface trap states in AlGaN/GaN Schottky-HEMT as well as MOS-HEMT but their results conclude that the trap states are mainly located at the surface interface within the dielectric or the passivation layer^12,13^. However, from a device point of view, characterization of the AlGaN/GaN interface would be more beneficial because this interface is directly related to carrier transport.

In this study, we tried to include all types of AlGaN/GaN interfacial trapping analysis for a better understanding. We extracted the interface trap density (*D*_*it*_) between AlGaN and GaN and the deep-level/border trap density *N*_bt_ in the AlGaN barrier layer in a long-channel AlGaN/GaN HEMT fabricated on a SiC substrate. We mainly focused on the trap states inside the AlGaN layer, located at the interface and near the interface of the AlGaN/GaN, and tried to eliminate other probable interfacial trap contributing factors such as dielectric layers for passivation. We used the conventional frequency-dependent C–V and G–V characteristics to understand the interactions of the interface traps^14,15^. Along with these characteristics, we also investigated the deep-level/border trap behavior in the accumulation region by examining split C–V characteristics, which are typically observed in the conventional Si MOS structure^16,17^. Although some researchers have discussed border/bulk trap extraction with threshold voltage shift profiling, discharging-based trap energy profile technique, etc., the frequency-dependent CV method for border trap density extraction for AlGaN/GaN heterostructure is not present^18–20^. We further performed low-frequency (1/*f*) noise measurements as they are a highly powerful tool for analyzing the defects and impurities in semiconductor devices and because they aid in the estimation of the efficiency and reliability of these devices^21^.

## Experimental details

**Figure 1:**
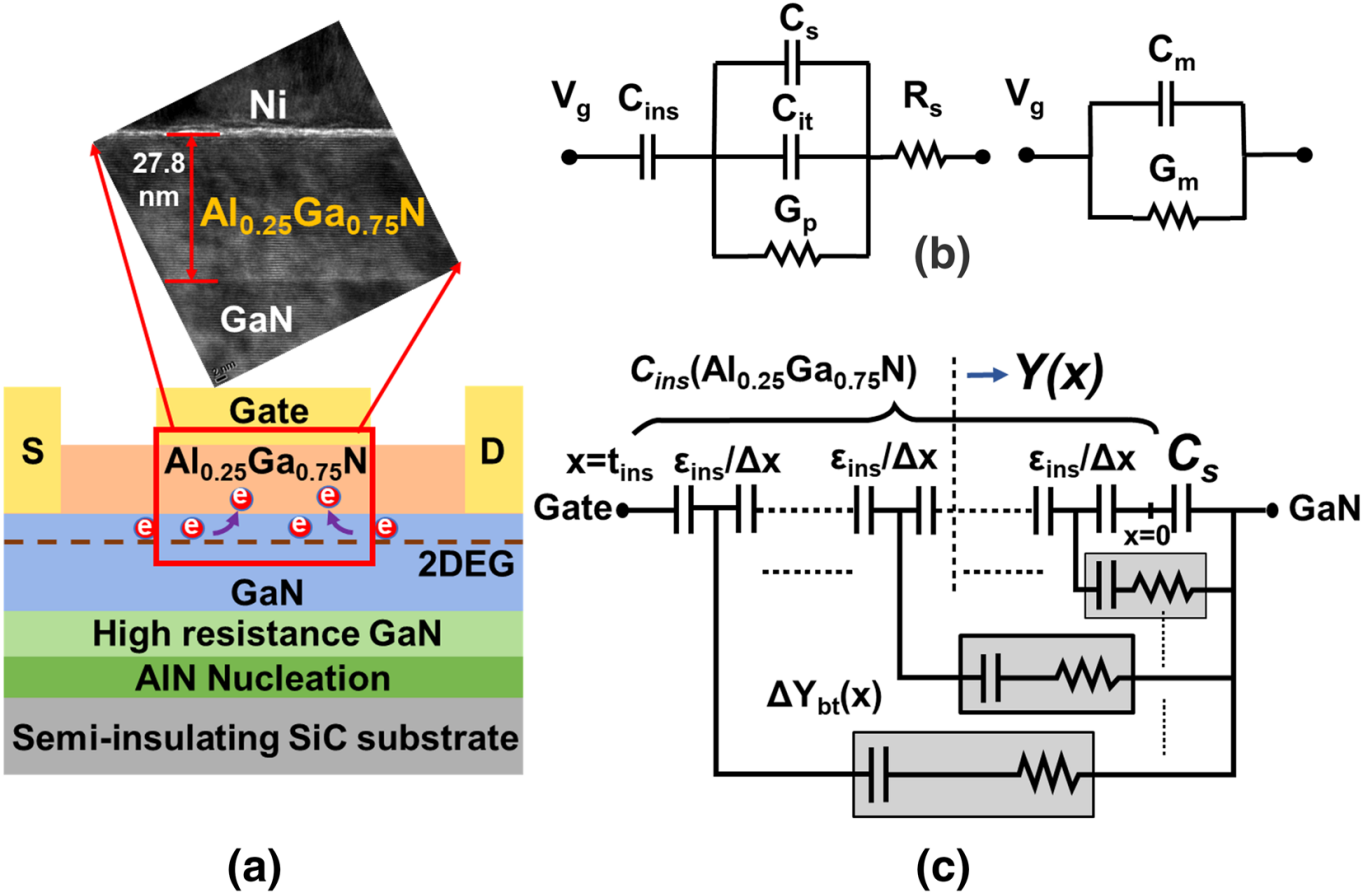
(**a**) Schematic cross-section and high-resolution TEM image of Al_0.25_Ga_0.75_N/GaN device. (**b**) Equivalent circuit diagram representing metal–insulator–semiconductor structure in depletion mode. (**c**) Equivalent circuit representing distributed bulk-oxide trap model^16,17^.

Figure 1a illustrates the cross-sectional schematic and the transmission electron microscopy (TEM) image of the HEMT device used in this study. Epitaxial layers were grown on a semi-insulating 330 µm SiC substrate by metal–organic-chemical-vapor-deposition (MOCVD). Layers were deposited from bottom to top in the following order: an AlN nucleation layer, a 2.6 µm high-resistance GaN layer, a 150 nm GaN channel, and a 28 nm Al_0.25_Ga_0.75_N barrier layer. Hall measurements revealed the mobility (*µ*_*n_Hall*_) and the sheet charge density (2DEG) to be 2200 cm^2^·V^−1^·s^−1^ and 9 × 10^12^ cm^−2^, respectively. Mesa isolation was carried out with Cl_2_ based inductively-coupled-plasma (ICP) etching to isolate the devices. Before ohmic metal deposition, the substrate was diluted with a mixture of HCl and deionized water (1:5) for 30 s to remove any kind of formed native oxide. To facilitate ohmic contact formation, a Si/Ti/Al/Ni/Au (1/25/160/40/100 nm) alloy was deposited on source and drain area using an electron beam (e-beam) evaporator and rapid thermal annealing at 830 °C was subsequently performed in N_2_ ambient for 30 s. The contact resistance (*R*_*C*_) and sheet resistance (*R*_*SH*_) were extracted by transmission-line-method (TLM) measurements to be 1.2 Ω·mm and 320 Ω/□ respectively. A Ti/Au (20/300 nm) padding layer was deposited using the E-beam evaporator to achieve strong probe contact. Finally, gate metal consisting of Ni/Au (20/400 nm) was also deposited using e-beam evaporator. The gate length (*L*_*g*_), gate width (*W*_*g*_), and source-to-drain distance (*L*_*sd*_) of the fabricated devices were 14, 50, and 18 µm, respectively. All the devices had the same source-to-gate (*L*_*sg*_) distance and gate-to-source distance (*L*_*gd*_) of 2 µm. From the high-resolution TEM image shown in Fig. 1a, the well deposited Al_0.25_GaN_0.75_N/GaN interface can be observed. The thickness of Al_0.25_GaN_0.75_N was well around 28 nm (27.8 nm) and formed a good interface with the GaN channel layer.

## Extraction model

### Interface trap model

In conventional Si MOSFET devices, the conductance method is one of the most popular methods for determining the interface trap density (*D*_*it*_)^14,22^. Typically, the interface between the dielectric and the semiconductor is analyzed for trap extraction. In our study, we applied the conductance method to our device structure. Because the Al_0.25_Ga_0.75_N barrier layer has a wide band gap (~ 4 eV) and a high dielectric constant (~ 9.4), it can act as an insulator and perform comparably to a dielectric material. The conductance method relies on the extraction of the equivalent parallel conductance (*G*_*P*_) from the measured frequency-dependent C–V and G–V characteristics. Figure 1b shows the equivalent circuit of a MOSFET in the depletion region, where *C*_*it*_, *C*_*S*_ and *R*_*S*_ represents the interface trap capacitance, the semiconductor capacitance, and series resistance, respectively. The interface trap capacitance can be expressed as *C*_*it*_ = *qD*_*it*_ (where *q* denotes the elemental charge). *G*_*P*_ can be determined by the following equation:

1 $$G_{p} = \frac{{\omega^{2} C_{ins}^{2} G_{c} }}{{G_{c}^{2} + \omega^{2} \left( {C_{ins} - C_{c} } \right)^{2} }}$$

Here, *C*_*ins*_ is the insulator capacitance; *ω* is the angular frequency; and *C*_*c*_ and *G*_*c*_ are, respectively, the corrected measured capacitance and conductance corresponding to the series resistance *R*_*S*_.

Using the normalized *(G*_*P*_*/ω)*_*max*_ value, we can determine the value of *D*_*it*_ as follows^23^:

2 $$D_{it} \approx \frac{2.5}{{Aq}}\left( {\frac{{G_{p} }}{\omega }} \right)_{\max }$$

Here, *A* denotes the device area.

The trap response of the interface states can be determined from the Shockley–Read–Hall statistics of capture and emission^24^:

3 $$\tau = \frac{1}{2\pi f} = \frac{1}{\omega } = \frac{{e^{{\left( {\frac{\Delta E}{{K_{B} T}}} \right)}} }}{{\sigma \nu_{th} D_{dos} }}$$

Here, Δ*E* denotes the difference of energy between the conduction band *E*_*C*_ and trap energy level *E*_*T*_. *K*_*B*_ and *T* are the Boltzmann constant and temperature. *σ*, *v*_*th*_ and *D*_*dos*_ represent the cross–section of traps, the average thermal velocity, and the effective density of states, respectively.

### Border trap model

The distributed circuit model shown in Fig. 1c was used for the extraction of border traps^16,17^. It can provide information on the border trap states inside the insulator bulk with frequency-dependent C-V measurement. This model can be represented by the following first-order differential equation:

4 $$\frac{\partial Y}{{\partial x}} = - \frac{{Y^{2} }}{{j\omega \varepsilon_{ins} }} + \frac{{q^{2} N_{bt} \ln \left( {1 + j\omega \tau } \right)}}{\tau }$$

This equation has a boundary condition of *x* = 0, *Y* = *jωC*_*S*_, while *Y* being the total admittance. *N*_*bt*_ in the above equation denotes the density of border traps in the insulator layer.

Usually, the carriers in the channel region and the border traps in the insulator layer can exchange charge through tunneling^16^. The average time (*τ*) required for an empty trap to capture electron can be calculated as^25^

5 $$\tau = \tau_{ \circ } e^{2kx}$$

$${\text{where,}}\;k = \frac{{\sqrt {2m* \times E_{b} } }}{\hbar }$$

Here, *τ*_*o*_ denotes the time constant of the capture and emission of a trap. *x* denotes the distance between the interface and the trap. m* and *k* denote the effective mass of the Al_0.25_Ga_0.75_N layer and the attenuation coefficient respectively. The barrier height between the Al_0.25_Ga_0.75_N and the GaN channel conduction band is denoted by *E*_*b*_ and the reduced Plank’s constant by *ħ*.

*τ*_*o*_ can be characterized as

6 $$\tau_{ \circ } = \left( {n_{s} v_{th} \sigma } \right)^{ - 1}$$

where *n*_*s*_, *v*_*th*_ and *σ* are the electron density of the channel surface, the average thermal velocity of the electron, and the border trap cross-section area of capture/emission, respectively.

Equation 4, can be simplified into the following equation for the total capacitance, *C*_*tot*_^26^,

7 $$C_{tot} = \frac{1}{{\frac{1}{{C_{ins} }} + \frac{1}{{2k\varepsilon_{ins} }}C_{2} (\omega )}}$$

8 $$C_{2} (\omega ) = 2k\sqrt {\frac{{\varepsilon_{ins} }}{{qN_{bt} }}} \coth (B) + \ln (\omega \tau_{o} ) - \coth^{2} (B)$$

9 $$B = a\tanh \left( {\sqrt {\frac{1}{{qN_{bt} \varepsilon_{ins} }}C_{s} } } \right) - \frac{1}{2k}\sqrt {\frac{{qN_{bt} }}{{\varepsilon_{ins} }}} \ln (\omega \tau_{o} )$$

Using *N*_*bt*_ and *τ*_*o*_ as fitting parameters, the best-fitted curve of *C*_*tot*_ with respect to frequency can be generated for the measured capacitance which will be discussed more in the results and discussion section.

The probing distance (*X*_*p*_) of a border trap with a fixed frequency (*f*) while, *ωτ* = 1, can be described as^27^

10 $$X_{p} = \frac{1}{2k}\ln \frac{1}{{2\pi f\tau_{ \circ } }}$$

## Results and discussion

**Figure 2:**
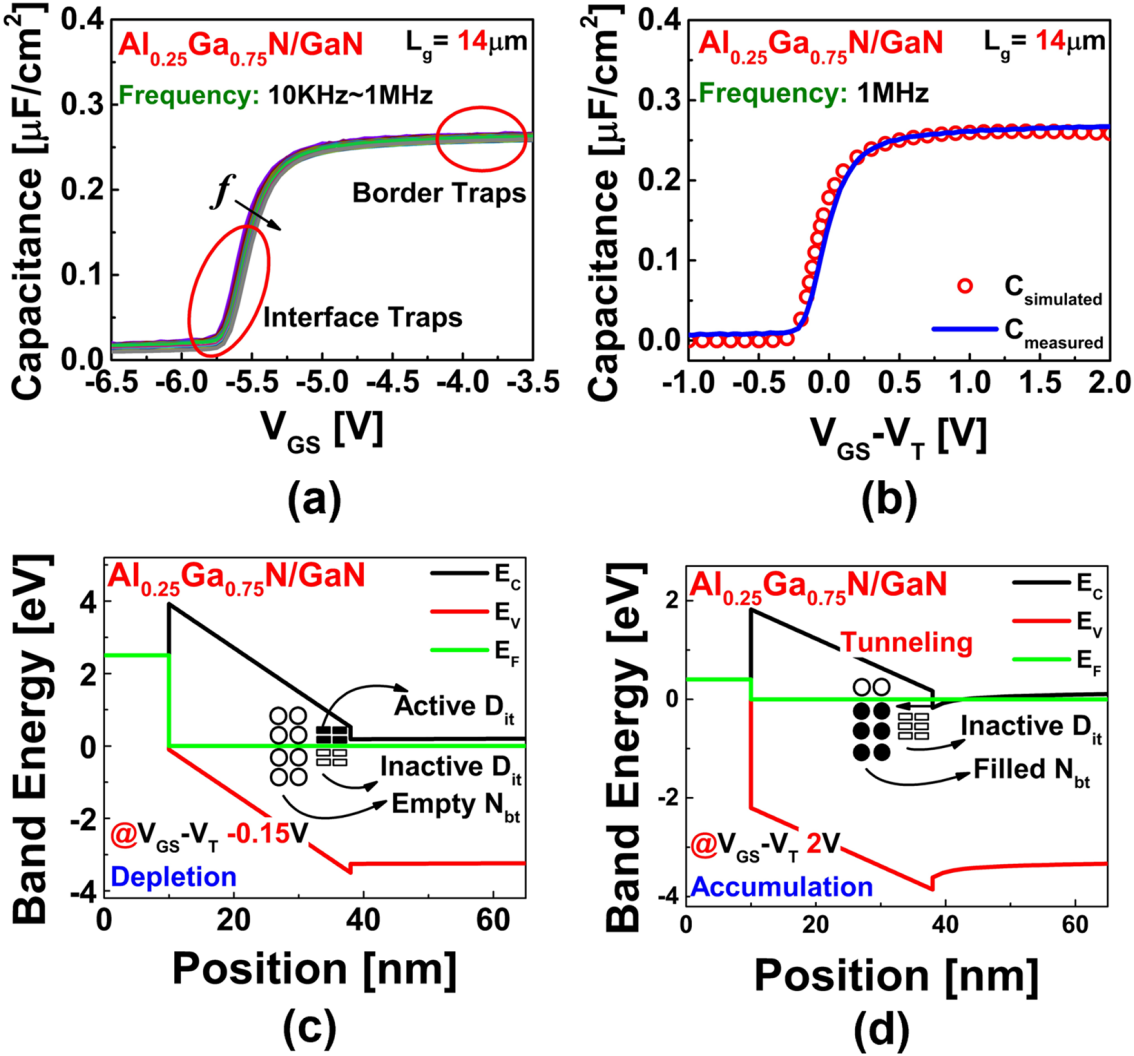
(**a**) Results of frequency-dependent C–V measurements showing active response region of traps. (**b**) Comparison between measured and simulated C–V characteristics. Simulated band diagram showing trap behavior (**c**) in depletion and (**d**) in accumulation.

Figure 2a shows the results of the frequency-dependent C–V measurements of the Al_0.25_GaN_0.75_N/GaN HEMT where frequency dispersion is evident. Frequency dispersion can be caused by various reasons. Some of the main causes of frequency dispersion during C–V measurement are parasitic effect, lossy interfacial layer, surface roughness, and quantum mechanical confinement etc^28^. Among them, the most influential reason is the lossy interfacial layer of AlGaN/GaN. The trap states in the AlGaN layer are mostly the reason behind the dispersion. The frequency dispersion in the depletion region indicates that this region is the dominant region for interface traps, whereas the dispersion in the accumulation region indicates the dominant region of the border traps. We used the Nextnano simulation tool (one-dimensional Poisson–Schrodinger solver) to compare the measured and simulated capacitance with respect to a constant gate overdrive (*V*_*GS*_ − *V*_*T*_), as shown in Fig. 2b. It is evident that both the measured and the simulated C–V curves are similar which indicates lower leakage current effect on the measured data. Thus, the AlGaN layer can be treated as an insulator owing to its high dielectric constant, similar to the MIS/MOS structure. Figure 2c, d show the band diagrams (determined via simulation) in the depletion and accumulation regions, respectively. In the depletion region, the interface traps above the Fermi level *E*_*F*_ are mostly active; this causes the capture and emission of the carriers in the channel region. In the accumulation region, where *E*_*F*_ penetrates the conduction band *E*_*C*_, the electrons on the surface are captured and emitted by the border traps via tunneling.

A Keysight B1500A semiconductor device analyzer and an Agilent 4384A precision LCR meter were used for all DC measurements. 1/*f* measurements were performed using a setup comprising battery operated SRS SR570 low-noise current preamplifier, HP 35670A dynamic signal analyzer, and a 1 Hz filter unit.

The insulator capacitance was determined by the following equation:

11 $$C_{ins} = \frac{{\varepsilon_{ \circ } \varepsilon_{ins} }}{{t_{ins} }}$$

**Figure 3:**
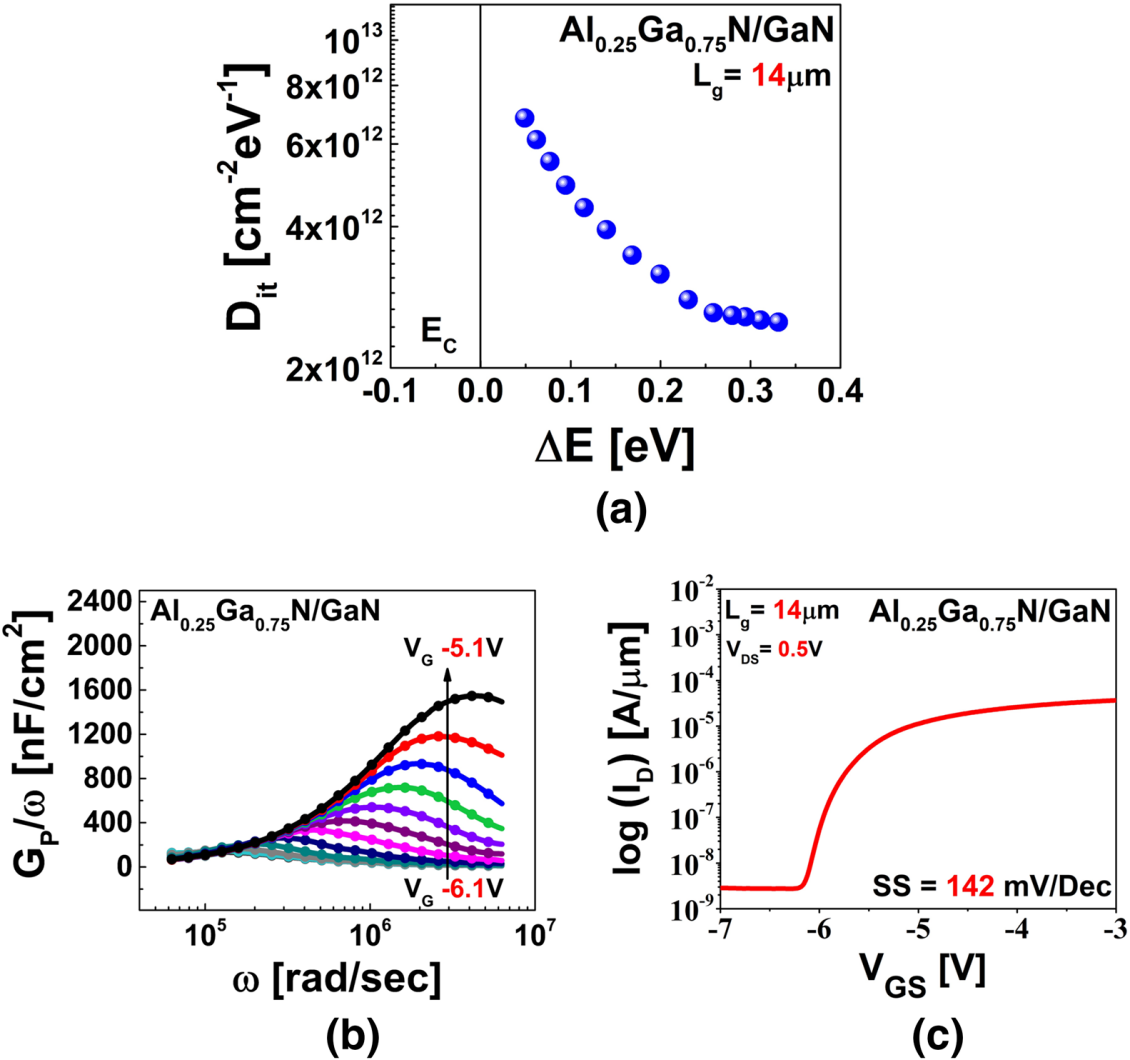
(**a**) Distribution of interface traps as a function of band energy state. (**b**) Equivalent parallel conductance (*G*_*P*_*/ω*) with respect to frequency at different gate bias points. (**c**) Basic transfer curve (log(*I*_*D*_)–*V*_*GS*_) showing SS of device.

Here, *ε*_*o*_ is the permittivity of free space and *ε*_*ins*_ is the relative permittivity of the Al_0.25_Ga_0.75_N layer. As it is known from the literature, the value of *ε*_*ins*_ as calculated from *ε* =  − 0.5*x* + 9.5—where *x* denotes the Al content of the Al_x_Ga_1−x_N layer—for *x* = 25% is around 9.375^29,30^. The tensor components of AlN and GaN’s {0001} relative permittivity are linearly interpolated to obtain the relation. The parallel equivalent conductance *G*_*p*_ was calculated using Eq. 1 with correction of the measured capacitance and conductance for the series resistance. Figure 3b shows a plot of the parallel conductance *G*_*p*_/*ω* versus the angular frequency *ω*. *D*_*it*_ was measured from the (*G*_*p*_/*ω*)_max_ peak, using Eq. 2; The extracted value of *D*_*it*_ using the conductance method was in the range of 2.5 × 10^12^ cm^−2^·eV^−1^ to 7.1 × 10^12^ cm^−2^·eV^−1^ which is well within the range of 10^11^–10^14^ cm^−2^ eV^−1^ for S-HEMT and MOS-HEMT from literature^10,12,31^. Figure 3a shows the active *D*_*it*_ with respect to the trap energy (Δ*E*), which was determined from Eq. 3. For this calculation, the frequency corresponding to (*G*_*p*_/*ω*)_max_ was considered. At room temperature (300 K), the average thermal velocity *v*_*th*_ and the effective density of states (*D*_*dos*_) of the GaN material were considered to be 2.6 × 10^7^ cm·s^−1^ and 1.2 × 10^18^ cm^−3^, respectively^31^. The value of the capture cross-section *σ* was assumed to be 3.4 × 10^−15^ cm^2^ from the literature^32^. The reliability of the extracted value of *D*_*it*_ was determined via a theoretical calculation of the subthreshold swing (SS) using the following equation^33^:

12 $$SS = \frac{kT}{q}\ln 10.\left( {1 + \frac{{qD_{it} }}{{C_{ins} }}} \right)$$

The value of the SS calculated from the lowest extracted *D*_*it*_ was around 143 mV·dec^−1^, whereas the value determined by the basic I–V measurement was found to be 142 mV·dec^−1^ (Fig. 3c). This similarity of the measured and calculated values confirms the reliability of the extracted value of *D*_*it*_.

**Figure 4:**
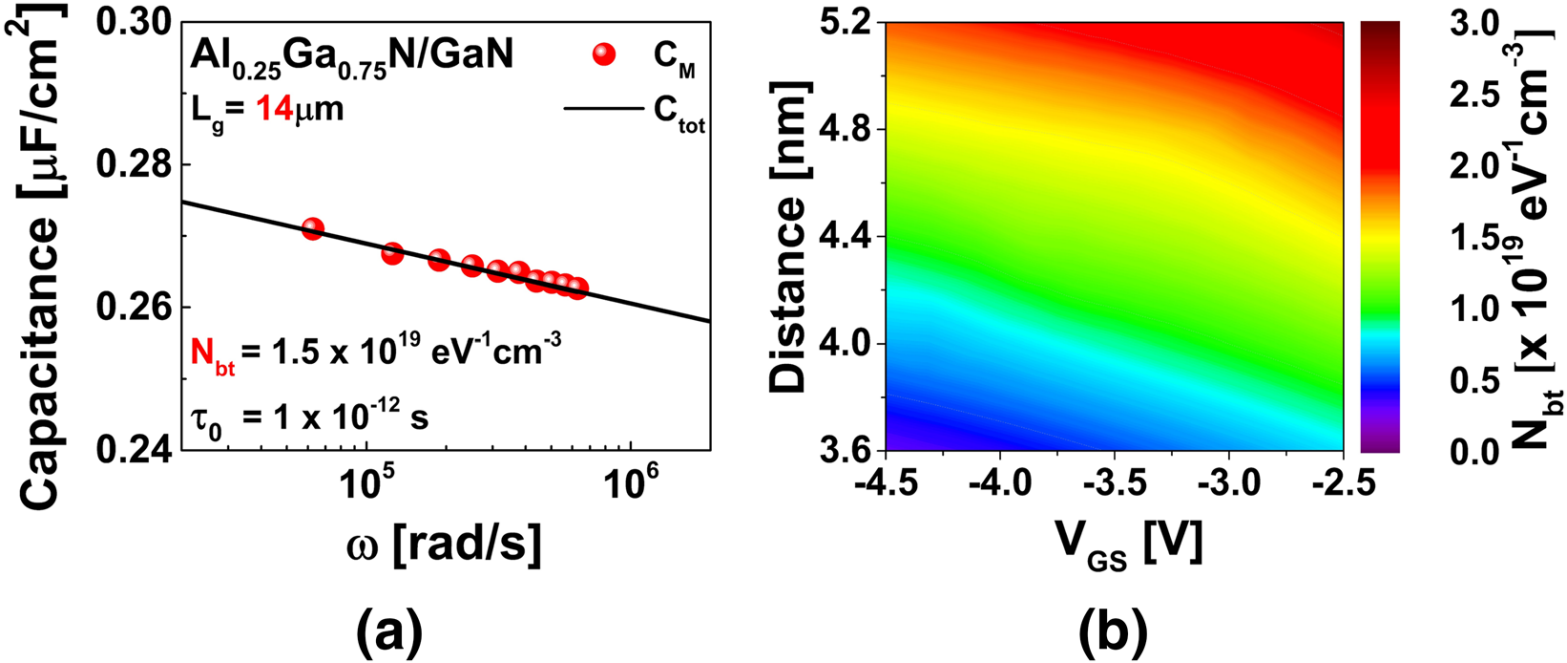
(**a**) Fitting curves generated using distributed circuit model at *V*_*GS*_ =  − 3.5 V. (**b**) Contour mapping of border trap distribution in Al_0.25_Ga_0.75_N layer from Al_0.25_Ga_0.75_N/GaN interface.

We used the parameters in Table 1 to extract the border trap density *N*_*bt*_. For the calculation of the attenuation coefficient, the effective mass of Al_0.25_Ga_0.75_N was considered to be 0.19*m*_*o*_ (where *m*_*o*_ denotes the electron mass at rest)^34^. The semiconductor capacitance *C*_*S*_ was estimated via Nextnano simulation at an accumulation gate bias of − 3.5 V, which was the primary *N*_*bt*_ extraction voltage considered in this study. From Eq. 4, the best-fitted capacitance curves were obtained at − 3.5 V under consideration of *N*_*bt*_ and *τ*_*o*_ as variable fitting parameters. The best-fitted curve was obtained at *N*_*bt*_ = 1.5 × 10^19^ cm^−3^·eV^−1^ and *τ*_*o*_ = 1 × 10^−12^ s, as shown in Fig. 4a. Here, *C*_*M*_ denotes the capacitances measured at various applied frequencies at − 3.5 V and *C*_*tot*_ represents the fitted curve. The spatial distribution of *N*_*bt*_ as a function of both the applied *V*_*GS*_ and the probing distance into the Al_0.25_Ga_0.75_N layer from the Al_0.25_Ga_0.75_N/GaN interface is shown in Fig. 4b. The *N*_*bt*_ values were extracted at various applied voltages at a particular applied frequency. The probing depth into the Al_0.25_Ga_0.75_N layer from the interface was calculated by Eq. 10 using different *τ*_*o*_ values associated with the *N*_*bt*_ values. Because the border traps exhibit more dominant characteristics at lower frequencies, we employed a low frequency of 10 kHz to extract the probing depth. With an increase in *V*_*GS*_, the Fermi level *E*_*F*_ tended to penetrate to a greater depth into the conduction band *E*_*C*_. As a result, more electrons tended to tunnel into the deep-level traps. As all parameters except *τ*_*o*_ were fixed, *τ*_*o*_ showed an inverse relation with the probing depth.

**Table 1:** Parameters used and extracted values of *D*_*it*_ and *N*_*bt*_.

Sample	Al_0.25_Ga_0.75_N/GaN
Parameter	Value
*t*_*ins*_ [nm]	28
*ε*_*ins*_	9.375
m* [*m*_*o*_]	0.19
*E*_*b*_ [eV]	0.8
*k* [nm^−1^]	1.99
*C*_*S*_ [µF·cm^−2^]	0.27
*τ*_*o*_ [s]	1 × 10^−12^
*D*_*it*_ [cm^−2^·eV^−1^]	2.5 × 10^12^
*N*_*bt*_ [cm^−3^·eV^−1^]	1.5 × 10^19^
*N*_*t*_ [cm^−3^·eV^−1^]	1.3 × 10^19^

**Figure 5:**
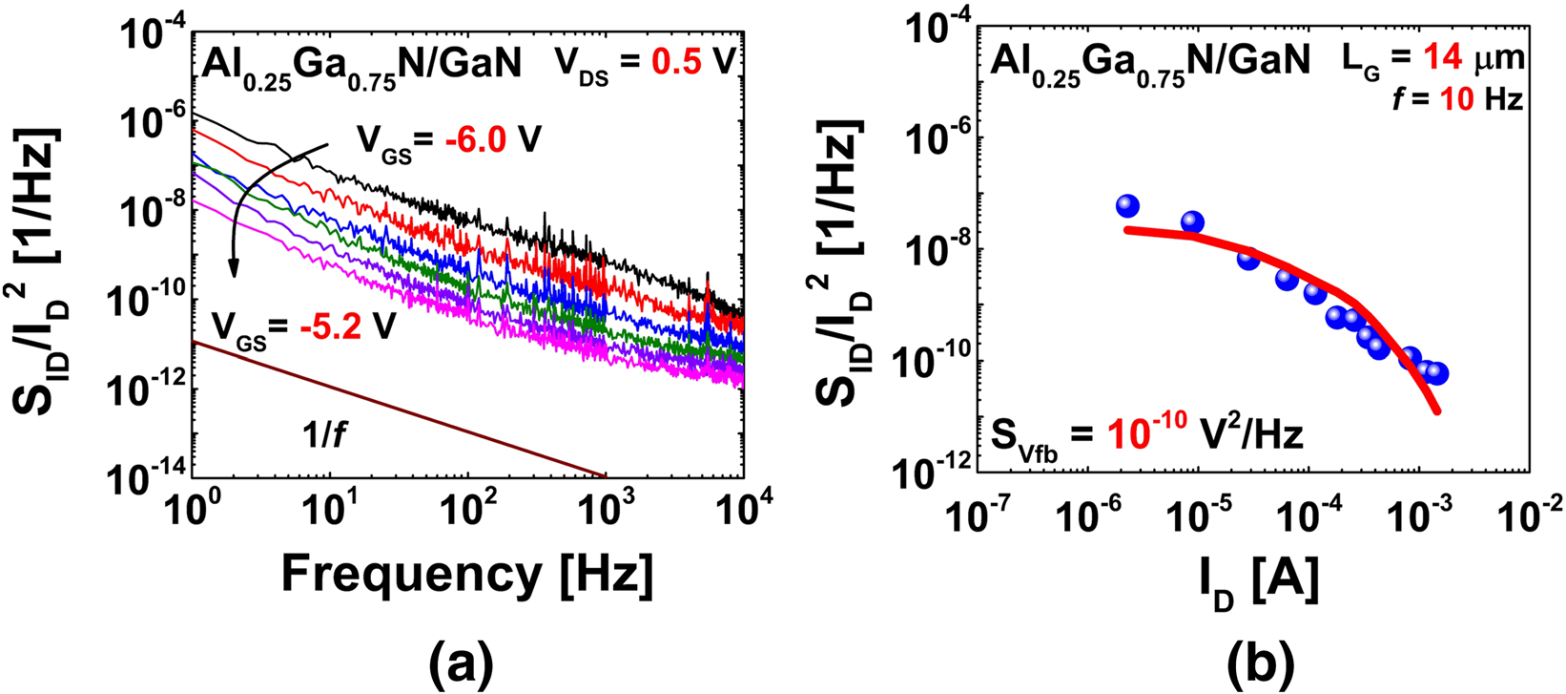
(**a**) Noise spectral density (*S*_*ID*_/*I*_*D*_^2^) with respect to frequency at various gate bias (*V*_*GS*_) points. (**b**) Noise spectral density (*S*_*ID*_/*I*_*D*_^2^) and (*g*_*m*_/*I*_*D*_)^2^ as functions of drain current *I*_*D*_.

1/*f* noise measurements were performed by varying the gate voltage *V*_*GS*_ and fixing the drain bias *V*_*DS*_ at 0.5 V. Figure 5a shows the normalized *S*_*ID*_/*I*_*D*_^2^ (drain current noise spectral density) with respect to frequency up to 10^4^ Hz under varying *V*_*GS*_ from the linear region. It is evident that as *V*_*GS*_ increased, and the device transitioned from weak inversion to strong inversion, noise level (*S*_*ID*_/*I*_*D*_^2^) decreases. Plotting of the normalized *S*_*ID*_/*I*_*D*_^2^ as a function of I_D_ (drain current) provided results that were more explanatory. Figure 5b shows a plot of the normalized *S*_*ID*_/*I*_*D*_^2^ (blue spheres) as a function of *I*_*D*_ at a frequency of 10 Hz. The channel carrier trapping phenomenon of the gate dielectric can be explained using the carrier number fluctuation (CNF) model by the following equations^21,35,36^:

13 $$\frac{{S_{ID} }}{{I_{D}^{2} }} = \left( {\frac{{g_{m} }}{{I_{D} }}} \right)^{2} S_{Vfb}$$

14 $$S_{Vfb} = \frac{{q^{2} N_{t} kT\lambda }}{{WLC_{d}^{2} f}}$$

Here, *S*_*Vfb*_ denotes the flatband voltage power spectral density; *kT*, the thermal energy; *WL*, the channel area; *C*_*d*_, the dielectric capacitance; *f*, the frequency; and *N*_*t*_, the bulk/border trap density. *λ* denotes the tunneling attenuation distance of the dielectric, which is expressed as λ = [4π(2 m*Ф_*B*_)^1/2^/*h*]^−1^, where *Ф*_*B*_ is the dielectric barrier height^37^. According to the CNF model, the terms *S*_*ID*_/*I*_*D*_^2^ and (*g*_*m*_/*I*_*D*_)^2^ vary in similar ranges with the drain current or gate voltage. From Fig. 5b, it is evident that both *S*_*ID*_/*I*_*D*_^2^ (blue spheres) and (*g*_*m*_/*I*_*D*_)^2^ (red line) vary similarly over several decades under varying *I*_*D*_. The *S*_*Vfb*_ value was calculated to be 10^−10^ V^2^·Hz^−1^ from Eq. 13. Using Eq. 14, we then calculated the border trap density *N*_*t*_ to be around 1.3 × 10^19^ cm^−3^·eV^−1^; this value is of a similar level to the values of the border trap density *N*_*bt*_ extracted from the distributed circuit model and well comparable to the data from literature of 10^18^–10^22^ cm^−3^ eV^−1^^20,36,38^.

## Conclusion

Unlike previous studies, which focused mainly on the insulator/AlGaN interface for trap extraction, the present study attempted to investigate the AlGaN/GaN interface for this purpose. We used modified versions of conventional MOS trap extraction methods to extract the interface trap density *D*_*it*_ and border trap density *N*_*bt*_ of the Al_0.25_Ga_0.75_N/GaN interface. We performed the extractions by considering the Al_0.25_Ga_0.75_N layer to be comparable to the insulator of the MOS structure on account of the relatively high dielectric constant of the former. The *D*_*it*_ value extracted by the conductance method was in the range of 2.5 × 10^12^ cm^−2^·eV^−1^ to 7.1 × 10^12^ cm^−2^·eV^−1^, and the *N*_*bt*_ value extracted using the distributed circuit model was 1.5 × 10^19^ cm^−3^·eV^−1^ with *τ*_*o*_ of 1 × 10^−12^ s. The border trap density *N*_*t*_ extracted using the CNF model via 1/*f* noise measurements was 1.3 × 10^19^ cm^−3^·eV^−1^ (same level as the extracted value of *N*_*bt*_), which confirmed the validity and reliability of our trap extraction method.

## Data Availability

The datasets generated during and/or analysed during the current study are available from the corresponding author on reasonable request.
