# Author Correction: A quantitative approach for trap analysis between Al_0.25_Ga_0.75_N and GaN in high electron mobility transistors

**DOI:** 10.1038/s41598-021-02854-3

**Published:** 2021-12-02

**Authors:** Walid Amir, Ju‑Won Shin, Ki‑Yong Shin, Jae‑Moo Kim, Chu‑Young Cho, Kyung‑Ho Park, Takuya Hoshi, Takuya Tsutsumi, Hiroki Sugiyama, Hideaki Matsuzaki, Tae‑Woo Kim

**Affiliations:** 1 Department of Electrical, Electronic and Computer Engineering, University of Ulsan, Ulsan, 44610 Korea; 2 Korea Advanced Nano Fab Center, Suwon, Gyeonggi‑do 16229 Korea; 3 NTT Device Technology Laboratories, NTT Corporation, Atsugi, Kanagawa 243‑0198 Japan

Correction to: *Scientific Reports* 10.1038/s41598-021-01768-4, published online 17 November 2021

The original version of this Article contained an error in the Acknowledgments section.

“This work was supported by the National Research Foundation of Korea (NRF) grant funded by the Korean government (MSIP; Ministry of Science, ICT and Future Planning, NRF- 2019R1A2C1009816 and NRF- 2019M3F5A1A01076973) and Nano Material Technology Development Program through the National Research Foundation of Korea (NRF) funded by the Ministry of Science, ICT and Future Planning (2009-0082580).”

now reads:

“This work was supported by a National Research Foundation of Korea (NRF) grant funded by the Korean government (MSIP; Ministry of Science, ICT and Future Planning, NRF- 2019R1A2C1009816 and NRF-2019M3F5A1A01076973).”

The original Article has been corrected.

